# Supplementary material for: Behavior Differentially Shapes Spontaneous Cortical Network Dynamics Across Frequencies
Source: bioRxiv. 2026 Jul 5:2026.07.05.736600. Preprint. [Version 1] doi: 10.64898/2026.07.05.736600 (PMC13345138; doi:10.64898/2026.07.05.736600)
Supplement: Supplement 1 [file NIHPP2026.07.05.736600v1-supplement-1.pdf]

bioRxiv preprint doi: https://doi.org/10.64898/2026.07.05.736600; this version posted July 6, 2026. The copyright holder for this preprint (which was not certified by peer review) is the author/funder, who has granted bioRxiv a license to display the preprint in perpetuity. It is made available under a CC-BY 4.0 International license.

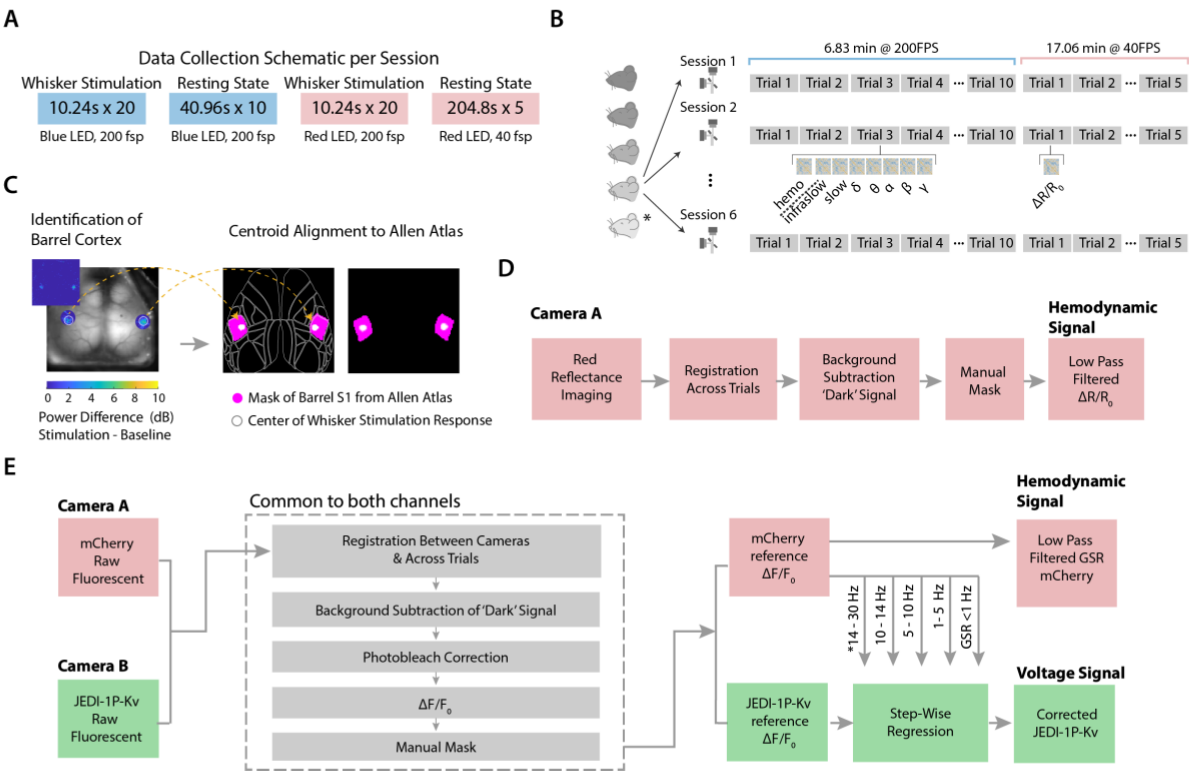

Supplemental Figure 1-1 | Data Collection and Preprocessing A) Schematic illustrating the different trial types recorded during one imaging session. Top row denotes the trial type which is either a whisker stimulation trial or resting state trial. Directly underneath in the colored boxes are the durations and number of trials recorded for each type. The color of the block denotes which LED was used. Underneath each colored block there is labeled the LED type and the frame rate used for that block of trials. **B)** The schematic extends on what is shown in panel A to highlight group-level data. Data from four mice consist of 6 resting state trials with both blue and red LEDs. For one mouse there is only blue LED data across 3 sessions that were twice as long, resulting in the same amount of net resting state data. **C)** Cross-session alignment to the Allen Atlas was performed using the whisker stimulation data. An image showing the power difference between stimulation and baseline was obtained for each whisker stimulation session. Pixels with increased power represent the whisker barrels (left), which define the two ROIs. Using centroid alignment these two ROIs are then co-registered to the centroid of a mask of barrel cortex from the Allen Atlas (right). **D)** Preprocessing steps applied to the red reflectance imaging from a single channel **E)**

bioRxiv preprint doi: https://doi.org/10.64898/2026.07.05.736600; this version posted July 6, 2026. The copyright holder for this preprint (which was not certified by peer review) is the author/funder, who has granted bioRxiv a license to display the preprint in perpetuity. It is made available under a CC-BY 4.0 International license.

Preprocessing pipeline for the dual camera image acquisition. Steps common to signals from both cameras are in gray boxes. Hemodynamic correction is applied using band-pass filtered versions of the mCherry signal that are regressed out of the JEDI-1P-Kv signal. The corrected JEDI-1P-Kv signal represents the voltage signal referenced throughout the paper. The simultaneously acquired hemodynamic signal refers to the low pass filtered global signal regressed mCherry signal.

bioRxiv preprint doi: https://doi.org/10.64898/2026.07.05.736600; this version posted July 6, 2026. The copyright holder for this preprint (which was not certified by peer review) is the author/funder, who has granted bioRxiv a license to display the preprint in perpetuity. It is made available under a CC-BY 4.0 International license.

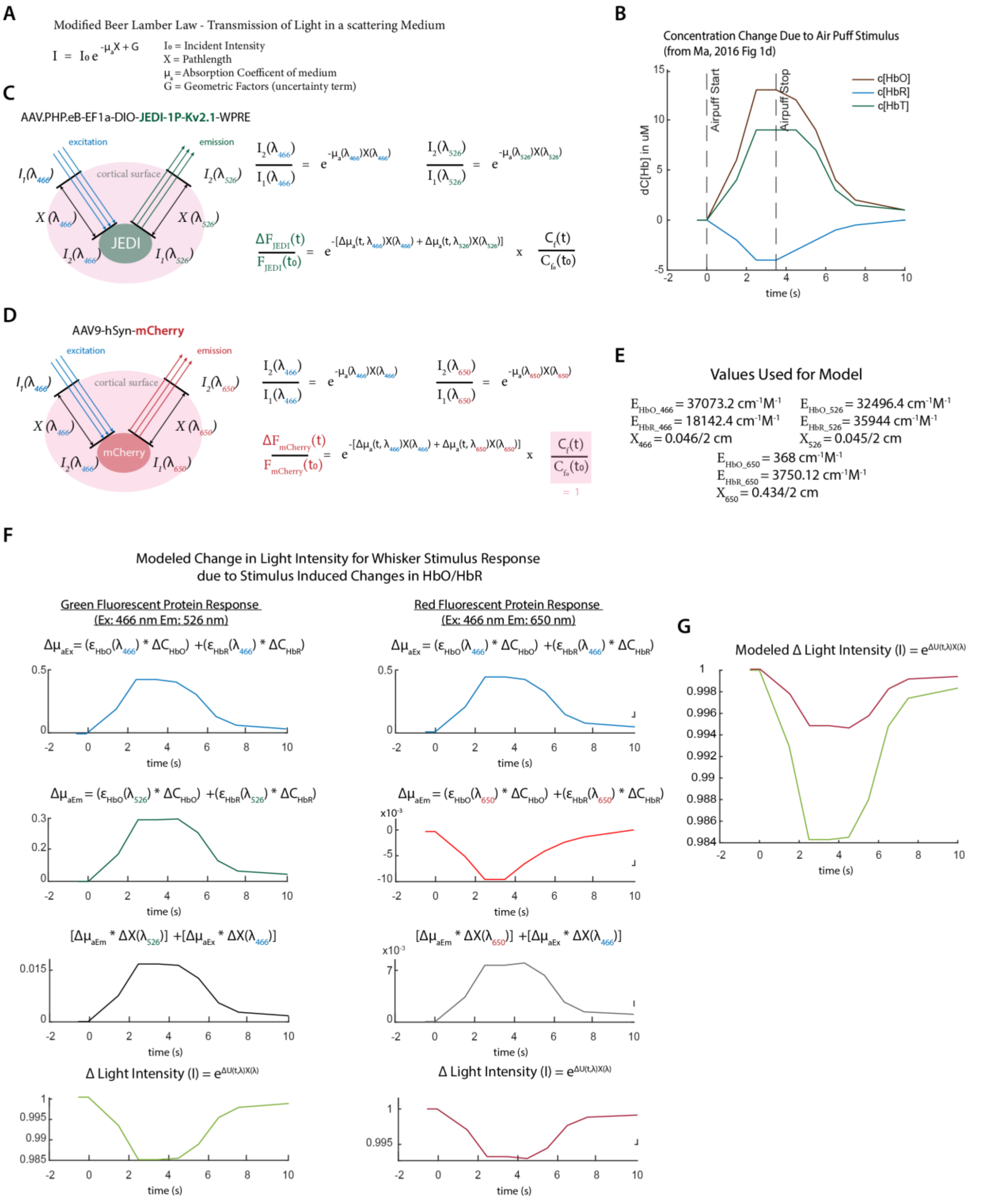

Supplemental Figure 1-2 | Modeled Hemodynamic Contributions to Fluorescent Signals A) Light traveling through a scattering medium like the brain can be modeled through the Modified Beer's Law. **B)** Changes in concentration in barrel cortex of HbO (brown), HbR (light blue) and HbT (green) for a whisker stimulus as reported in [20]. **C)** Schematic of variables to consider for fluorescent imaging of a GFP such as JEDI-1P-Kv (left) and mathematical formulation (right) to describe change in measured light intensity based on a formula in panel A. **D)** Same as in panel

bioRxiv preprint doi: https://doi.org/10.64898/2026.07.05.736600; this version posted July 6, 2026. The copyright holder for this preprint (which was not certified by peer review) is the author/funder, who has granted bioRxiv a license to display the preprint in perpetuity. It is made available under a CC-BY 4.0 International license.

C but for a RFP like mCherry. **E)** Modeled values from the literature for the extinction coefficients for HbO and HbR, along with the reported values for optical pathlength for all 3 wavelengths of interest [20]. **F)** Left column represents the modeled change in light intensity for JEDI-1P-Kv using the values using the formula in panel C and the values from panel B and E. From top to bottom are the modeled changes in light for: excitation wavelength, emission wavelength, both combined with factoring in the pathlength traveled, the final measured change in light intensity. Right column represents the same thing but for mCherry. Note the difference in magnitude on the y-axis for the excitation and emission wavelengths of mCherry. There is a stronger absorption due to HbO and HbT at the excitation wavelength. **G)** An overlay of the modeled change in light intensity due to neurovascular coupling that would be observed for both JEDI-1P-Kv (green) and mCherry (red) during the whisker stimulus response. For both fluorescent signals a negative change in fluorescence due to neurovascular coupling is expected.

bioRxiv preprint doi: https://doi.org/10.64898/2026.07.05.736600; this version posted July 6, 2026. The copyright holder for this preprint (which was not certified by peer review) is the author/funder, who has granted bioRxiv a license to display the preprint in perpetuity. It is made available under a CC-BY 4.0 International license.

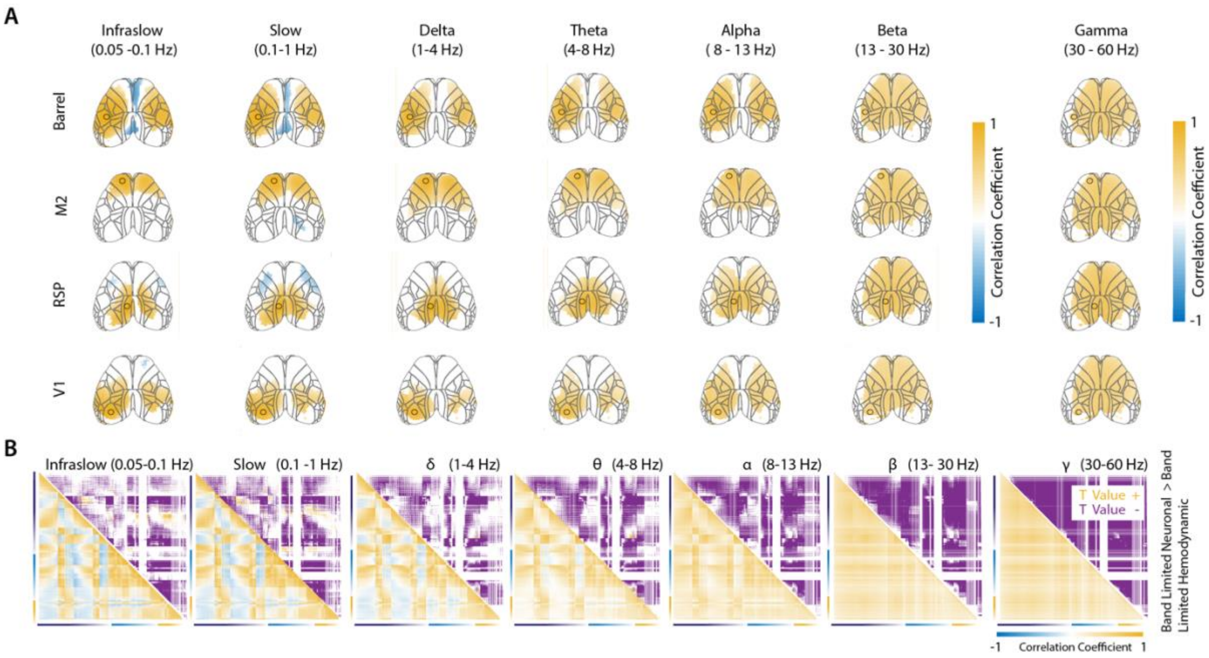

Supplemental Figure 2-1 | Different Bandlimited Functional Connectivity Across Faster Frequency Bands in mCherry Signal A) Average static seed-based FC for bandlimited mCherry signal processed and filtered in the same way as the corrected JEDI signal. Pixels are thresholded to pixels with significant connectivity as determined using a two-sided t test corrected for multiple comparisons with FDR. **B)** Bottom left half represents the average static FC matrix for the bandlimited mCherry signals**.** Top right is the two-sample t-test results comparing the bandlimited neuronal FC to that from the bandlimited mCherry FC. Thresholded t values are shown. Corrected for multiple comparisons using FDR.

bioRxiv preprint doi: https://doi.org/10.64898/2026.07.05.736600; this version posted July 6, 2026. The copyright holder for this preprint (which was not certified by peer review) is the author/funder, who has granted bioRxiv a license to display the preprint in perpetuity. It is made available under a CC-BY 4.0 International license.

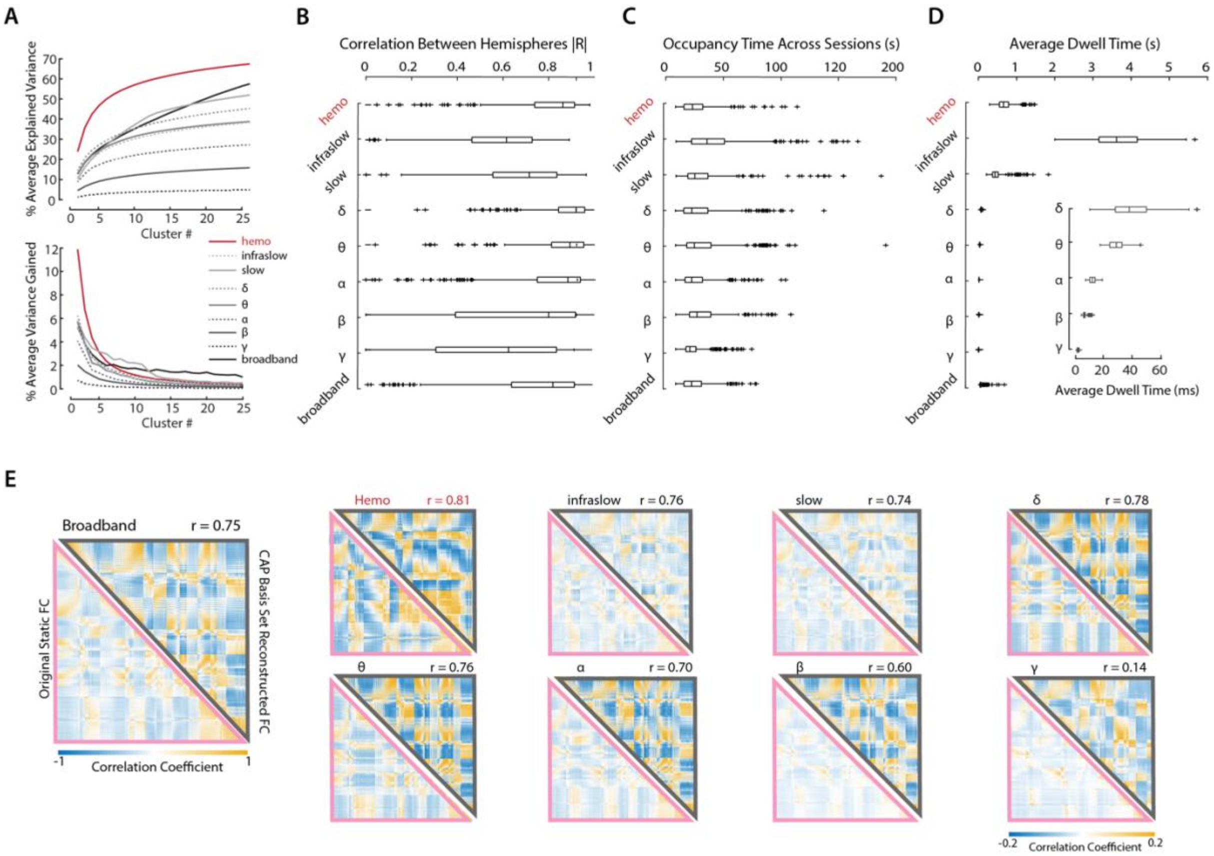

Supplemental Figure 3-1 | CAP Properties Across Frequency Bands A) Session average change in average explained variance and variance gained as a function of increasing cluster number K across different signal types and frequencies. **B)** Distribution of the absolute value of the correlation between left and right hemispheres. Sorted as a function of frequency bands. **C)** Distribution of the average percentage of all imaging frames assigned to each CAP as a function of different frequencies. **D)** Distribution of the dwell time in seconds, which represents how much time is spent in a given CAP state, plotted as a function of different frequencies. The bottom excerpt shows the same dwell time in units of ms for theta through gamma band frequencies. **E)** Bottom left pink triangle represents the average FC matrix obtained from the raw data. This is the same data as in Figure 1G shown here to highlight a comparison to the top triangle. Top grey triangles represent the average matrix obtained from reconstructing session-level data based on individually selected ideal k CAPs. The spatial correlation between the two matrices is displayed to the right of each square. Represented is the data for all frequency bands and signal types.

bioRxiv preprint doi: https://doi.org/10.64898/2026.07.05.736600; this version posted July 6, 2026. The copyright holder for this preprint (which was not certified by peer review) is the author/funder, who has granted bioRxiv a license to display the preprint in perpetuity. It is made available under a CC-BY 4.0 International license.

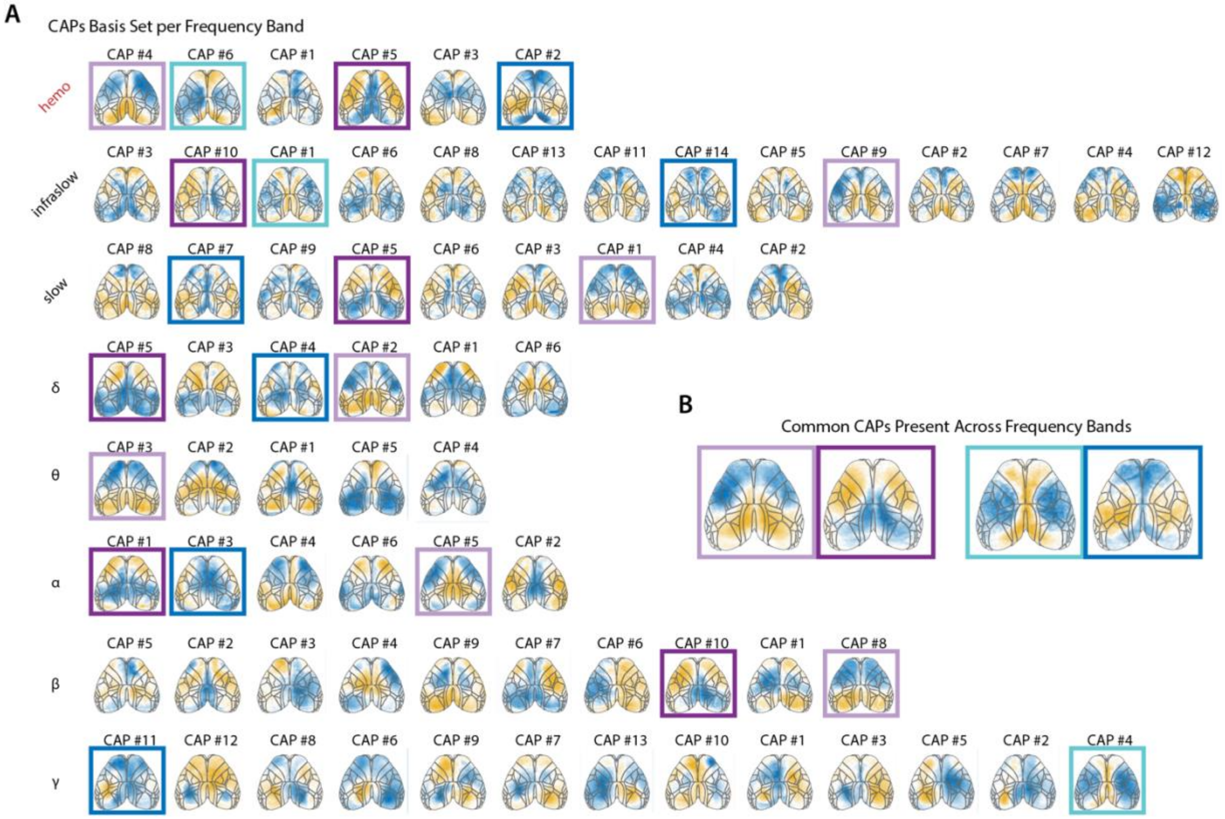

Supplemental Figure 4-1 | CAP Basis Set Across Frequencies A) The basis set obtained from clustering all the single session frequency specific CAPs with a cutoff of 0.8. Colors underneath certain CAPs highlight repeated spatial patterns that are present across frequency bands **B)** Images obtained from averaging the CAPs highlighted with the respective colors. These images represent the average spatial patterns that are present across frequencies. These 4 average images are grouped into two anticorrelated pairs.

bioRxiv preprint doi: https://doi.org/10.64898/2026.07.05.736600; this version posted July 6, 2026. The copyright holder for this preprint (which was not certified by peer review) is the author/funder, who has granted bioRxiv a license to display the preprint in perpetuity. It is made available under a CC-BY 4.0 International license.

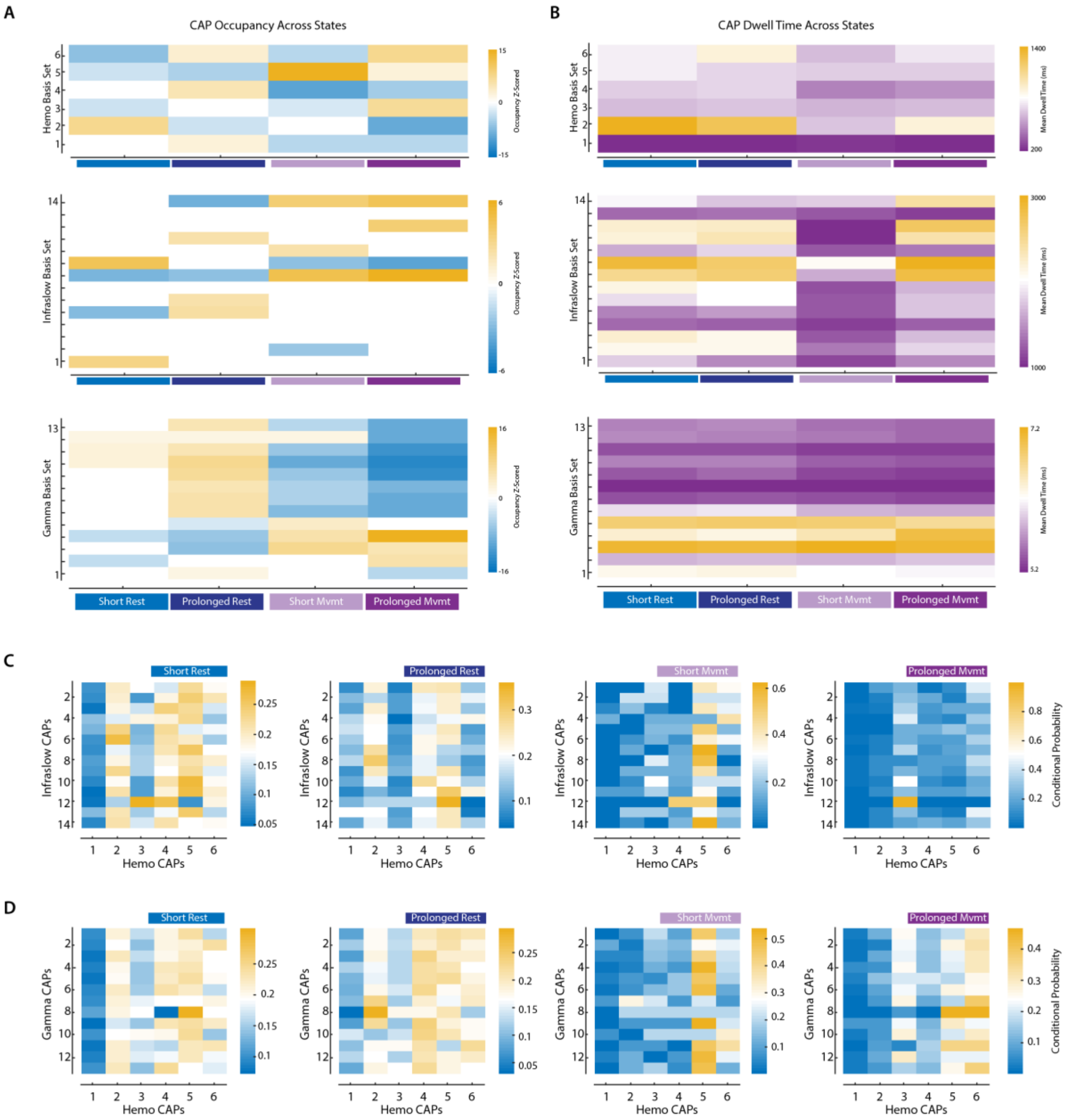

Supplemental Figure 5-1 | CAP Occupancy and Dwell Time Across States A) Same as Figure 5D, but for the three other signals that were consistent across CAP cutoffs. **B)** Same as Figure 5E but for the three other signals that were consistent across CAP cutoffs.
